# The mechanisms of milder clinical symptoms of COVID-19 in children compared to adults

**DOI:** 10.1186/s13052-024-01587-z

**Published:** 2024-02-14

**Authors:** Caiyin Luo, Wanwen Chen, Junying Cai, Yuwen He

**Affiliations:** Department of Pharmacy, the First Affiliated Hospital of Guangzhou Medical University, 28 Qiaozhong Middle Road, Liwan District, 510120 Guangzhou, China

**Keywords:** Children, COVID-19, Clinical symptoms, Mechanism, Immune response

## Abstract

In stark contrast to adult patients, children who contract Severe Acute Respiratory Syndrome Coronavirus 2 (SARS-CoV-2) typically manifest milder symptoms or remain asymptomatic. However, the precise underlying mechanisms of this pathogenesis remain elusive. In this review, we primarily retrospect the clinical characteristics of SARS-CoV-2 infection in children, and explore the factors that may contribute to the typically milder clinical presentation in pediatric Coronavirus Disease 2019 (COVID-19) patients compare with adults patients with COVID-19. The pathophysiological mechanisms that mitigate lung injury in children are as follows: the expression level of ACE2 receptor in children is lower; the binding affinity between ACE2 receptors and viral spike proteins in children was weaker; children have strong pre-activated innate immune response and appropriate adaptive immune response; children have more natural lymphocytes; children with COVID-19 can produce higher levels of IgM, IgG and interferon; children infected with SARS-CoV-2 can produce lower levels of IL-6 and IL-10; children have fewer underlying diseases and the lower risk of worsening COVID-19; children are usually exposed to other respiratory viruses and have an enhanced cross-reactive immunity. Comprehending the relative contributions of these processes to the protective phenotype in the developing lungs can help in the diagnosis, treatment and research pertaining to children with COVID-19.

## Background

The World Health Organization declared COVID-19, a newly emergent coronavirusis, is caused by SARS-CoV-2 [1]. A large number of studies have shown that children with COVID-19 tend to experience less severity symptoms and lower mortality rates than adults. Multiple consolidated case reports of hospitalized children with COVID-19 from China [2, 3], Italy [4], Spain [5] and the United States [6] have indicated that children make up a relatively small percentage (typically<2%) of patients who present with clinically recognized symptoms of SARS-CoV-2 infection. In addition, infections in the majority of children were mild with a substantial proportion (5–21%) showing no symptoms at all, while another large group displayed symptoms similar to that of a typical viral upper respiratory tract infection [4, 7]. Although a small number of children who have recently contracted SARS-CoV-2 have developed multisystem inflammatory syndrome, the proportion of severe cases ranges from 1 to 6% [8], and the mortality rate remains relatively low (0-0.2%) [9]. An extensive study by Grant et al., which collected data from 24,410 adults diagnosed with COVID-19 across 9 countries, revealed that 17% of patients required hospitalization (comprising 7,504 patients from 33 studies) and recorded a 7% mortality rate (involving 10,402 patients from 73 studies) [10]. By utilizing previously collected data, Khera et al. conducted a comparative analysis of clinical symptoms of children and adults infected with SARS-CoV-2. The result demonstrated a lower incidence of common COVID-19 symptoms in pediatric patients [11]. Specifically, the study revealed that among infected children, 59.9% (compared to 80% in adults) exhibited fever, 55.9% (as opposed to 84% in adults) presented with cough, and 20% (in contrast to 38.4% in adults) had a runny nose. This review provides an overview of the current understanding of the differences between children and adults infected with SARS-CoV-2, with a particular focus on the influence factors contributing to mild clinical symptoms observed in children with COVID-19.

## Clinical features of children with SARS-CoV-2 infection

A majority of children with COVID are asymptomatic or present with mild clinical symptoms. According to the World Health Organization’s living guidelines, the severity of COVID-19 is classified into mild, moderate, severe and critical. Mild cases are those with symptoms of upper respiratory tract infection but no pneumonia [1]. A population study conducted in Iceland observed a lower incidence of cases among children under 10 years of age, compared to those over 10 [12]. Of the 17,877 children who had reported symptoms to the United States Centers for Disease Control (US CDC), fever (46%), cough (37%), headache (15%), diarrhea (14%), and sore throat (13%) were the most commonly observed symptoms in children aged ≤ 9 years, while children aged 10–19 years commonly exhibited headache (42%), cough (41%), fever (35%), myalgia (30%), sore throat (29%), shortness of breath (16%), and diarrhea (14%) [13]. This indicates that the symptoms of COVID-19 in children over 10 years old are different from those in children under 10 years old. Another study conducted at Wuhan Children’s Hospital reviewed 171 children with confirmed COVID-19 and provided more detailed symptom information. The most frequently reported symptoms in these children were cough (48.5%), pharyngeal erythema (46.2%), and a fever of at least 37.5 °C (41.5%). Among those with fever, 32.1% had a fever above 38 °C, with most falling within the range of 38.1 to 39.0 °C [14]. Fever is the most common presenting symptom among children, and less common symptoms include diarrhea, fatigue, rhinorrhea, vomiting, anosmia, and dysgeusia. Notably, gastrointestinal symptoms such as abdominal pain, nausea, vomiting, and diarrhea were often the initial manifestations seen in children upon infection with SARS-CoV-2 [15, 16]. Additionally, they tend to recover more quickly than infected adults and typically recover within 1–2 weeks of the onset of the illness [17]. While fever and cough are the main clinical symptoms observed in both children and adults infected with SARS-CoV-2, the incidence of fever tends to be higher in children than that in adults. On the other hand, cough is more common in adults [18].

## The possible factors that affect the presence of mild symptoms in children with COVID-19

### Children have an effective pre-activated innate immune response and appropriate adaptive immune response

The main reason for the milder symptoms and lower mortality rate in children infected with SARS-CoV-2 compared with adults is thought to be the age-related differences in innate and adaptive immune responses. When children are infected with SARS-CoV-2, they generate specific immunopathological responses that encompass both innate immunity, such as type I interferon (IFN-I) and pro-inflammatory cytokines, and adaptive immunity, including the production of neutralizing antibodies against virus-specific antigens by plasma cells. Autoimmunity potential also plays a role in these responses, with outcomes in affected children being influenced by the activation of immune cells and imbalances in various cytokines [20]. In cases where the innate immune response is only mildly suppressed and the subsequent adaptive immune response is both timely and appropriate, it can help control the infection leading to the occurrence of mild disease. Conversely, a delayed innate immune response not only promotes unrestricted SARS-CoV-2 replication, but also results in a prolonged and intensified compensatory immune response. Liu et al. suggested that this difference in severity may be due to the varying immune responses of adults and children to the virus [19]. Numerous studies have consistently demonstrated age-related differences in both the innate and adaptive immune responses [20], indicating that the immune system in children may respond differently to COVID-19 compared to adults. Age-related changes in the immune system are pervasive and affect nearly every aspect of its function, typically resulting in pronounced pro-inflammatory responses that intensify with age [21]. Children tend to have a higher level of innate immune response than adaptive immune response. This pre-activated innate immune system helps clear SARS-CoV-2 infection and may prevent an excessive immune response [22]. On the contrary, in adult patients, an intense adaptive immune response may occur, leading to the development of conditions such as acute respiratory distress syndrome and associated inflammation [23, 24]. In addition, in a study conducted by Loske et al., nasal swab samples were collected from individuals in two distinct groups: patients infected with SARS-CoV-2 and healthy controls ranging in age from 4 to 77 years [25]. Analysis of single cell sequencing data revealed that healthy adults had considerably fewer immune cells in their nasal samples compared to healthy children, who presented with diverse immune cell subsets, mostly neutrophils. Collectively, these studies have shown that it is the functional levels of innate and adaptive immunity, particularly those related to T cell amount and function, such as an overly vigorous antigen-specific antibodies response, low levels of IFNs and high levels of interleukins, that may contribute to the severity of COVID-19.

### Children have more lymphocytes than adults

Primarily produced in the lymphoid organs, lymphocytes are crucial components of the immune system and play a vital role in defending the body against viral infections. CD4 + T lymphocytes produce potent cytokines that serve to activate the immune system and promote the production of antibodies by B lymphocytes. On the other hand, CD8 + T lymphocytes are responsible for destroying virus-infected cells, thereby reducing the viral load and limiting the spread of virus [26]. Recent studies have indicated that T cell lymphopenia in both blood and respiratory fluid can serve as a marker of severe COVID-19 in both children and adults [27, 28]. The output of lymphocytes from thymus is highest at around one year of age, and gradually decreases thereafter until it becomes negligible at approximately 85 years of age, due to the dynamic changes in T cell development throughout life. In children between the ages of 5 days and 5 years, lymphocytes account for 60–65% of the total white blood cells, while in adults, lymphocytes account for 30–35% of the total white blood cells. Thus, it can be inferred that children have more lymphocytes than adults. A study that compared the immune responses of 65 children and younger adults up to 24 years of age to 60 adults hospitalized with COVID-19 revealed that absolute lymphocyte counts were significantly higher in pediatric patients [27]. The study conducted by Fang et al., also found that in contrast to the reduced lymphocyte count typically observed in adults, the white blood cell count and absolute lymphocyte count were mostly normal in children with COVID-19, with no signs of lymphocyte depletion [29]. These researches suggested that this may be linked to the relatively underdeveloped immune system in children, which can result in a lower level of adaptive immunity. Severe COVID-19 patients often experience lymphocytopenia, and lower lymphocyte counts are correlated with a longer time for negative nucleic acid conversion of SARS-CoV-2 and higher levels of organ damage indicators. Because children have higher lymphocyte counts than adults, they are less susceptible to lymphocyte depletion, resulting in fewer severe cases in children than in adults [30]. Children have more natural T cells in their bodies than adults. When exposed to SARS-CoV-2, children’s natural T cells recognize the virus and quickly eliminate it. The similarities observed between T cell dysfunction in aging adults and in those with severe COVID-19 suggest that the gradual onset of T cell dysfunction in adulthood may contribute to the impaired T cell response associated with severe COVID-19 [31]. From above evidences, it can be speculated that age-related differences in T cell amount and function likely contribute to the higher incidence of severe COVID-19 in adults compared to children.

There is evidence to suggest that the frequency of lymphocyte subsets, particularly CD3+, CD4+, and CD8 + T cells, may have an inverse correlation with disease severity in COVID-19. Jiang et al. reported that patients with COVID-19 exhibit significant depletion in CD3+, CD4+, CD8 + T cells, with the depletion in CD8 + T cells being more pronounced [32]. Deng et al. reported significantly lower CD3+, CD4+, and CD8 + T cell counts in patients with severe COVID-19 compared to patients without severe COVID-19 [33]. Sun et al. found that CD8 + T cell count was lower in patients with severe and critical disease and could serve as an independent predictor of disease severity [34]. Fang et al. observed that a decrease in CD4 + and CD8 + T cell subsets in adult patients diagnosed with COVID-19 in Wuhan based on T lymphocyte subsets analysis [29]. However, in contrast to the previous studies mentioned, a study by Argun et al. has shown that lymphocyte subsets including CD4 + T cells, increased in children with COVID-19 [35]. Based on these data on lymphocyte subsets, it is suggested that children with SARS-CoV-2 may develop a more protective immune response against the virus compared to adults. This may limit the spread of the virus in the body and preventing an excessive immune response, leading to absent or limited systemic inflammation.

### Children infected with COVID-19 produce higher levels of IgM and IgG

In parallel with a robust cellular immune response to SARS-CoV-2, children are also capable of mounting a strong serologic response to mild SARS-CoV-2 infections [36]. The disparities in the serologic response to SARS-CoV-2 between children and adults coincide with the recognized age-associated differences in B cell development. There are two types of memory B cells- CD27^dull^ and CD27^bright^- with the former being primarily responsible for producing immunoglobulins M (IgM). CD27^dull^ cells are the primary memory B cells found in children and they have the ability to produce high levels of IgM when exposeded to neoantigens. Native IgM is capable of recognizing and modifying autoantigens as well as neutralizing viruses through identification of the endogenous “danger signal” that is present in the viral envelope and imprinted in the membranes of infected and stressed cells [37]. IgM plays a crucial role in the initial stage of infection by recognizing and efficiently eliminating both SARS-CoV-2 and infected cells thus providing protection against COVID-19. Due to this mechanism, children may exhibit comparatively milder symptoms of the disease [38].

Immunoglobulins G (IgG) antibody is a key “neutralizing” antibody can recognize the spike protein on the surface of SARS-CoV-2 and block virus entry into cells. A study showed significant age differences in antibody responses to SARA-CoV-2 and the immune response is strongest in people 10 years of age and younger [39]. The results showed that the median IgG antibody level in 32 children aged 1 to 10 years was nearly five times higher than that in 127 adults aged 19 to 24 years. According to Weisberg et al. antibody responses of children and adults differ following an infection with SARS-CoV-2 [40]. A study conducted on 231 children and 1168 adults revealed that the serological responses in children are achieved through the targeting of distinct viral epitopes, as compared to adults [41]. Children with COVID-19 exhibited higher level of antibodies against the spike protein, which was the most frequently targeted protein by the immune response in children. Conversely, the most common antibody target in adults did not include the spike protein and adults have a greater abundance of antibodies [42]. The above studies indicate that adults who have contracted SARS-CoV-2 may produce an overly vigorous antibody response, whereas children infected with the virus are capable of generating potent SARS-CoV-2-specific IgG, which exhibits greater specificity against the virus compared to adults.

### Children infected with COVID-19 produce a greater amount of interferon

Interferon (IFN) is a cytokine that is produced by monocytes and lymphocytes, and it plays an crucial role in the body’s antiviral defense mechanism. It has been found that, in comparison to adults, the upper respiratory tract of children exhibit higher quantities of innate immune cells and pattern recognition receptor expression. As a result, children infected with SARS-CoV-2 appear to produce a greater amount of interferon and facilitate a stronger innate immune response in the initial stages of infection than their adult counterparts [43]. Children’s upper respiratory mucosal immune system is prone to a pre-activated state compared to adults, which enables their immune system to quickly release interferon and effectively counteract the spread of infection during the early phases of SARS-CoV-2 infection.

Type I interferon release plays a crucial role in innate immunity as it works against viral infections and triggers the activation of the adaptive immune response [44]. The innate immune response mediated by type I IFN and its associated functional signaling pathway is the crucial first step of host antiviral defense against acute SARS-CoV-2 infection [45]. The work conducted by Bastard et al. revealed that 13% (135/987) of patients with severe COVID-19 had autoantibodies that attacked type I IFN, whereas no such antibodies were detected in asymptomatic or mildly-affected patients or healthy individuals [46]. Likewise, Hadjadj et al. further demonstrated that the highly damaged type I IFN response in patients with severe and critical COVID-19, indicated by the absence of IFN-β and reduced expression of IFN-α [47]. In addition, reduced type I and III IFN responses have been demonstrated in severe and fatal cases of COVID-19 [48]. At present, studies have revealed variations in the type I IFN signal between children and adults who have been infected with SARS-CoV-2. Adults with severe COVID-19 have been found to have lower circulating level of type I IFN compared to children with COVID-19, which can be attributed to the production of neutralizing antibodies against type I IFN [46], as well as the decrease in both the gene expression [49] and the secretion of type I IFN by plasmacytoid dendritic cells (pDCs) [47]. Impaired innate immunity, mediated by the reduction of type I IFN signaling pathways, can lead to severe pneumonia in adults compared to children.

Furthermore, the accumulation of exogenous factors that reduce the IFN response is commonly observed with age. This may include chronic viral infections, like human cytomegalovirus, which can cause plasmacytoid DCs exhaustion and IFN secretion reduction, as well as the production of autoantibodies against IFN [46]. In addition, previous studies have demonstrated that impaired IFN response are associated with old age [50], obesity [51] and atherosclerosis [52], which may contribute to the observed age-related differences in severity of COVID-19 disease. Therefore, the decreased IFN signaling was mainly observed in patients over 65 years of age, which could potentially explain the higher incidence of severe COVID-19 in middle-aged and elderly adults.

### Children infected with COVID-19 produce fewer levels of IL-6 and IL-10

A cytokine storm is a characteristic of severe COVID-19 cases and is recognized as a clinical predictor of disease outcome and mortality [53]. Cytokine storm refers to systemic inflammation and markedly high level of proinflammatory cytokines in plasma—refers to the excessive production of proinflammatory cytokines in the plasma, causing systemic inflammation — These cytokines include tumor necrosis factor (TNF)-α, interleukin (IL)-1, IL-2, IL-6, IL-8, IL-12, IFN-α, IFN-β, IFN-γ, monocyte chemotactic protein 1 (MCP-1), and chemokines [chemokine (C-C motif) ligand (CCL1, CCL3, CCL10], which are rapidly produced in large quantities and can attack the host immune cells. Cytokine storm can be an significant contributing factor to the development of acute respiratory distress syndrome (ARDS) [54]. Excessive secretion of cytokines can lead to an increase in vascular permeability at the site of lesions and accelerate vascular exosmosis, making it easier for pathogens to enter the blood vessels. This can eventually result in diffuse injury to target cells such as pulmonary capillary endothelial cells and alveolar epithelial cells, leading to the development of ARDS, septic shock, multiple organ failure (MOF), and potentially, even death [55]. Existing studies indicate that an imbalance of cytokines is related to severe progression of pneumonia and subsequent tissue damage [56]. In a retrospective study of 41 COVID-19 cases conducted by Huang et al. at Zhongnan Hospital of Wuhan University, it was found that severe patients have significantly higher expression levels of proinflammatory factors [IL-2, IL-7, IL-10, granulocyte colony-stimulating factor (G-CSF), interferon-induced protein 10 (IP-10), MCP-1, macrophage inflammatory protein 1 α (MIP-1α)、TNF-α] in their plasma compared to mild parents. This indicates severe patients may experience ta cytokine storm [57].

Similarly, a study conducted on patients with severe COVID-19 revealed that the levels of IL-2, IL-6, IL-10, and TNF-α were significantly higher compared to non-severe patients [58]. In regard to children, Massalska et al. suggest that they are prone to less inflammatory cytokine production in response to viral infection due to their immature immune system and low adaptive immune response when compared to adults [59]. A study conducted by Jia et al. has also shown that children with mild COVID-19 exhibit cytokine characteristics similar to those of healthy children, indicating low level of inflammation [60]. Therefore, the symptoms of most children infected with SARS-CoV-2 tend to mild. A previous study has shown that cytokine storm induced by different viruses is not completely identical. For example, infection with Middle East Respiratory Syndrome Coronavirus (MERS-CoV) increases the levels of IFN-γ, TNF-α, and IL-17, while infection with SARS-CoV-2 leads to increased levels of IL-6, IL-12, and IFN-γ. Further, it has been confirmed that increased levels of IL-6 and IL-10 are independent and significant predictors of the severity and mortality of COVID-19 [61, 62].

As a multifunctional cytokine, IL-6 is secreted by various cells, including lymphocytes, endothelial cells, and monocytes, and plays an important role in the body’s immune response against infections [63]. Numerous studies indicate that cytokine storm, characterized by high levels of inflammatory cytokines, including IL-6, is a contributing factor in the progression and severity of COVID-19 [64]. It has been supported by a large number of research data that the serum concentration of IL-6 significantly increases in patients, especially those with severe symptoms, following infection with SARS-CoV-2 [65, 66]. A study in New York showed that the elevated level of IL-6 was positively correlated with bilateral and interstitial lung damage, indicating that IL-6 serve as both an independent and important predictor of the severity and mortality associated with COVID-19, as well as one of the most reliable indicators of prognosis and survival [67]. According to another meta-analysis comprising nine studies, patients with severe COVID-19 had a significantly higher mean concentration of IL-6 was than those with non-severe symptoms (mean difference of 38.6ng/L). Additionally, the analysis showed that elevated IL-6 levels were strongly related to a higher mortality rate [62]. Patients with IL-6 levels exceeding 55ng/L were found to be at an increased risk of severe COVID-19, in while those with IL-6 > 80ng/L surpassing a greater risk of mortality. These studies indicate that IL-6 levels serve as a reliable predictor of disease severity and clinical outcomes for COVID-19 parents.

IL-10, produced by newborn B cells and activated B cells, exerts an anti-inflammatory effect by reducing macrophage activationand the release and activity of inflammatory cytokines such as IL-6, TNF-α, and IL-1β. The production of IL-10 decreases with age [68]. It has been found that adult COVID-19 patients in the intensive care unit (ICU) exhibit a significantly higher plasma level of IL-10 compared to those not in the ICU [56]. Neumann et al. consider that IL-10 may play a role in promoting the production of systemic inflammatory cytokines and stimulating the activation and proliferation of T lymphocytes in patients with COVID-19 [69]. Li et al. tested cytokines in 30 children diagnosed with COVID-19 in Wuhan Children’s Hospital and found elevated IL-10 levels in 10 children, with a slight elevation in 6 children (less than twice the maximum normal reference value) and a significant IL-10 elevation in 2 children (more than three times the maximum normal reference value) [61]. Out of the two children with significantly elevated IL-10 levels, one developed severe COVID-19. Based on the above data, it can be concluded that IL-10 levels may be linked to the severity of COVID-19, as adaptive changes in IL-10 level leading to relatively mild pneumonia symptoms in children as compared to adults. To sum up, the increase in IL-10 level is related to the severity of COVID-19, making it a potential biomarker for track in the progression of the disease.

### Children have lower ACE2 receptor expression levels and weaker binding ability with spike protein

Upon binding of the receptor binding domain (RBD) of SARS-CoV-2 spike protein binds to the angiotensin-converting enzyme II (ACE2) receptor located on the upper respiratory tract mucosa [70], the virus can enters host cells via endocytosis or membrane fusion (penetration) [71]. Compared to adults, the expression of ACE2 receptors is lower, which may lead to the milder clinical symptoms in children. It has been shown that the expression of ACE2 begins to increase during late childhood (about 10 years of age), potentially providing a protective effect against the most aggressive pattern of the infection in younger children. Bunyavanich et al. performed single-cell RNA sequencing of nasal epithelial cells from 305 individuals between the age of 4 to 60 [72]. These patients were categorized based on age into four groups: younger children (<10 years old, *n* = 45), older children (10–17 years old, *n* = 185), young adults (18–24 years old, *n* = 46) and adults (≥ 25 years old, *n* = 29). The study established a linear regression model with the expression of ACE2 gene as the dependent variable and age groups as the independent variable. The results showed that ACE2 gene expression was lowest in younger children (mean log2 counts per million, 2.40) and increased with age, with mean log2 counts per million of 2.77 in older children (*n* = 185), 3.02 in young adults (*n* = 46), and 3.09 in adults (*n* = 29). Recent studies showed that there is positive correlation between age and the expression level of ACE2 in alveolar cells. Compared to adults, children have lower expression of ACE2 receptors in the lungs and nasal epithelium [73]. In addition, ACE2 expression vary among different organs in children, with higher expression in the lung and lower expression in the nasal epithelium. This limited virus entry may play a certain protective role in children [74].

Furthermore, Schouten et al. suggested that smoking would increase ACE2 expression, potentially leading to enhance the entry of coronavirus into lung epithelial cells [75]. Undoubtedly, smoking is more prevalent among adults compared to children, this may could increase the risk of severe symptoms in adults infected with SARS-CoV-2.

As mentioned above, the pathogenesis of human coronavirus mainly depends on the interaction between the spike protein of the virus and the transmembrane ACE2 receptors. Therefore, a molecular mechanism has been hypothesized to explain why children may be less susceptible to SARS-CoV-2 infection [39]. Fang et al. speculated that children may be less sensitive to SARS-CoV-2, due to the maturity and functions (such as binding ability) of ACE2 receptors in children are lower than those in adults [29]. Consistent with this conjecture, Heurich et al. suggested that the immune system of children is not yet fully developed, and the function of ACE2 receptors is low or relatively weak compared to adults, which leads to a limited entry route of virus and the inability to cause strong cytokine storm, explaining why most cases in children are mild [76].

### Adults with underlying diseases will increase the risk of COVID-19 becoming severe

Patients with underlying diseases are more susceptible to developing severe symptoms of COVID-19. Major risk factors for COVID-19 patients include hypertension, diabetes, cardiovascular disease, chronic obstructive pulmonary disease, and cerebrovascular disease [77, 78]. These underlying diseases can lead to abnormal immune response such as a “cytokine storm” triggered through epigenetic and metabolic reprogramming of immune activation, further promoting the progression of severe COVID-19, organ damage, and ultimately critical conditions for patients affected by the disease [79]. In patients with one or more comorbidities, the lymphocyte ratio was significantly lower than in those without comorbidities [80]. Overall, higher levels of inflammation indicators and longer hospitalization durations were observed in patients with comorbidities, suggesting that these patients might have higher inflammation levels, increased disease severity in SARS-CoV-2 infection, and require a longer time to recover. Among 171 children treated at Wuhan Children’s Hospital, three (1.8%) required intensive care, and all of them had underlying diseases, such as hydronephrosis, leukemia undergoing chemotherapy, and intussusception [81]. This demonstrates that children with underlying diseases are tended to progress to severe and critical cases. Since Children rarely have underlying diseases, such as cerebrovascular disease, diabetes, obesity, chronic obstructive pulmonary disease or cardiovascular diseases, compared to adults, the clinical symptoms of children infected with COVID-19 are milder.

For example, Xia et al. studied 82 COVID-19 patients, including 29 with hypertension (the hypertension group) and 53 without hypertension (the nonhypertension group) [82]. The hypertension group had a mortality rate of 20.7%, while the nonhypertension group had a rate of 9.4%. Compared with nonhypertensive patients, hypertensive patients exhibited higher neutrophil counts, serum amyloid A, C-reactive protein, and NT-proBNP as well as lower lymphocyte counts and eGFR. It indicates that COVID-19 patients with hypertension are at an increased risk of severe inflammatory reactions, serious internal organ injury, and disease progression [83]. The reason for that is as follows: after infection with SARS-CoV-2, the ACE2 level was found to be reduced due to binding with the spike protein of SARS-CoV-2 [84], suggesting that SARS-CoV-2 may reduce the level of ACE2 in infected cells, resulting in an imbalance between ACE1 and ACE2. The renin-angiotensin II-aldosterone axis, traditionally recognized as a key regulator of blood pressure, could become imbalanced in individuals with hypertension, leading to more serious organ injury. Accordingly, hypertension might be the underlying cause of increased susceptibility to more severe COVID-19.

A meta-analysis of retrospective studies has confirms that COPD is associated with a dramatically increased risk of disease progression in COVID-19 patients. COVID-19 patients with COPD had a 5.9-fold higher risk of progression compared to those without COPD. This increased risk could be due to viral infections in COPD patients, where systemic inflammation increases and symptom improvement is slow [85]. A study shows that the risk of COVID-19-related mortality is significantly and independently related to the preceding level of hyperglycaemia in people with type 1 and type 2 diabetes, and in type 2 diabetes the gradient of this risk association is steeper in people younger than 70 years than in those aged 70 years or older. It is possible that hyperglycaemia impairs host defences, including granulocyte and macrophage function [86]. Heart disease and diabetes increased the risk of death 20 times more than other risk factors [87]. Li et al. also reported that the mortality rate of patients with hypertension, cerebrovascular disease, and diabetes was two, three, and two times higher, respectively, in patients admitted to ICU [88]. Patients with previous cardiovascular metabolic diseases may face a greater risk of developing severe condition. Conversely, COVID-19 can also exacerbate heart damage. Additionally, the risk of infection increases with a higher body mass index (BMI). The results of a systematic study have confirmed that obesity is associated with an increased risk of death in COVID-19 patients [89]. These studies have shown that COVID-19 patients with underlying conditions have a higher risk of death compared to COVID-19 patients without comorbidities.

### Pre-exposure to other viruses enhances cross-reactive immunity of children

Patel et al. propose that one of the reasons for the milder clinical symptoms observed in children infected with SARS-CoV-2 might be their prior exposure to seasonal coronaviruses (such as HCoV-229E, HCoV-HKU1, HCoV-NL63 and HCoV-OC43) at a higher frequency than adults. This frequent exposure enhances cross-reactive immunity in children [20]. Compared to adults, children are usually affected by other respiratory viruses like respiratory syncytial virus (RSV), adenovirus (ADV), influenza virus, and rhinovirus during the winter [90], resulting in a higher level of antiviral antibodies in children’s bodies [91]. Children’s susceptibility to seasonal viruses is primarily due to the developing state of their immune systems, resulting in relatively lower immunity, which provides an opportunity for pathogenic microorganisms such as viruses to enter. However, this frequent exposure to various viruses also primes children’s immune systems, allowing them to respond quickly to SARS-CoV-2. In a study by Ng et al., the proportion of cross-reactive anti-SARS-CoV-2 spike protein antibodies was compared between adults and children who had not been exposed to the virus. In this study, it was discovered that 62% of children had cross-reactive antibodies that effectively neutralized SARS-CoV-2 infection in vitro, whereas only 5% of the adults exhibited the same response [92]. Consiglio et al. found an absence of IgG antibodies against HCoV-HKU1 and β coronaviruses among children with multisystem inflammatory syndrome (MIS-C) contrasted with high prevalence in healthy controls, raising the possibility that a prior exposure to seasonal coronaviruses might offer protection to children infected with SARS-CoV-2 from the development of MIS-C [93].

## Conclusions

There is compelling evidence demonstrating variations in the immune responses of children to COVID-19 compared to adults. The majority of children either experience mild symptoms or remain asymptomatic. Several hypotheses have been put forward to elucidate the lower incidence and less severe symptoms of SARS-CoV-2 infection in children relative to adults. These hypotheses encompass factors such as lower expression of ACE2 [72], weaker binding affinity between ACE2 and the virus spike protein, diminished intensity of the immune response, disparities in lymphocyte profiles, heightened capacity for natural antibody production, distinct patterns of inflammatory responses, interactions between different viruses in the respiratory tract (including the concurrent presence of other respiratory viruses in the respiratory mucosa), and numerous other facets [38].

## Data Availability

Not applicable.

## Abbreviations

ACE2: Angiotensin-Converting Enzyme II
ADV: Adenovirus
ARDS: Acute Respiratory Distress Syndrome
BMI: Body mass index
CCL: Chemokines (C-C motif) Ligand
COVID-19: Coronavirus Disease 2019
DCs: Dendritic Cells
G-CSF: Granulocyte Colony-Stimulating Factor
ICU: Intensive Care Unit
IFN-I: Type I Interferon
IgG: Immunoglobulin
IgM: Immunoglobulin M
IL: Interleukin
IP-10: Interferon-induced Protein 10
MCP-1: Monocyte Chemotactic Protein 1
MERS-CoV: Middle East Respiratory Syndrome Coronavirus
MIP-1α: Macrophage Inflammatory Protein 1α
MIS-C: Multisystem Inflammatory Syndrome in Children
MOF: Multiple Organ Failure
RBD: Receptor Binding Domain
RSV: Respiratory Syncytial Virus
SARS-CoV-2: Severe Acute Respiratory Syndrome Coronavirus 2
TNF: Tumor Necrosis Factor
US CDC: United States Centers for Disease Control
